# Sexual system, reproductive cycle and embryonic development of the red-striped shrimp *Lysmata vittata*, an invader in the western Atlantic Ocean

**DOI:** 10.1371/journal.pone.0210723

**Published:** 2019-01-15

**Authors:** Douglas Fernandes Rodrigues Alves, Laura S. López Greco, Samara de Paiva Barros-Alves, Gustavo Luis Hirose

**Affiliations:** 1 Departamento de Biologia, Universidade Federal de Sergipe–UFS, São Cristóvão, Sergipe, Brazil; 2 Programa de Pós-Graduação em Ecologia e Conservação, Universidade Federal de Sergipe–UFS, São Cristóvão, Sergipe, Brazil; 3 Universidad de Buenos Aires, CONICET, Instituto de Biodiversidad y Biología Experimental y Aplicada (IBBEA), Facultad de Ciencias Exactas y Naturales, Departamento de Biodiversidad y Biología Experimental, Laboratorio de Biología de la Reproducción y el Crecimiento de Crustáceos Decápodos, Buenos Aires, Argentina; 4 Laboratório de Ecologia de Ecossistemas Aquáticos, Universidade Federal de Uberlândia–UFU, Uberlândia, Minas Gerais, Brazil; Tokat Gaziosmanpasa University, TURKEY

## Abstract

Several decapod crustaceans are invaders, but little is known about the biological characteristics that potentiate the success of these decapods in invaded ecosystems. Here, we evaluate and describe some aspects of the reproductive biology and development of *Lysmata vittata*, an invasive shrimp species in the Atlantic Ocean. In addition, we intend to provide important insights into the biology of invasion by comparing the reproductive traits of this shrimp with some of the predictions about aquatic invasive species. We used experimental and laboratory observations to evaluate the functionality of protandric simultaneous hermaphroditism (PSH), the macro and microscopic development of the ovarian portion of the ovotestes, the reproductive cycle, and the embryonic development of *L*. *vittata*. We confirm the functionality of PSH in *L*. *vittata*. This shrimp has a rapid reproductive cycle; the ovarian portion of the ovotestes develops (mean ± SD) 6.28 ± 1.61 days after spawning. Embryonic development also occurs over a short time, with a mean (± SD) of 8.37 ± 0.85 days. The larvae hatch without macroscopically visible yolk reserves. Our study provides evidence that the invasive shrimp *L*. *vittata* has reproductive and embryonic developmental characteristics (i.e., short generation time and high reproductive capacity) that may be favorable to the establishment of populations during invasive processes.

## Introduction

Several decapod crustaceans are invaders, e.g., *Eriocheir sinensis* H. Milne Edwards, 1853 [1]; *Charybdis japonica* (Milne-Edwards, 1861) [2]; *Charybdis hellerii* (A. Milne-Edwards, 1867) [3–5]; *Callinectes sapidus* Rathbun, 1896 [6,7]; *Neocaridina davidi* (Bouvier, 1904) [8]. However, little is known about the biological characteristics that potentiate the success of these decapods in invaded ecosystems. Understanding the biological characteristics that facilitate the establishment of populations outside their natural limits could be used to predict future invasions. Some hypothesized attributes of successful aquatic invaders, including decapods, are: 1) abundant and widely distributed within their original range; 2) wide environmental tolerance; 3) high genetic variability; 4) short generation time; 5) rapid growth; 6) early sexual maturity; 7) high reproductive capacity; 8) broad diet (opportunistic feeding); 9) gregariousness; 10) natural mechanisms of rapid dispersal; and 11) being commensal with human activity (e.g., ship ballast-water transport) [9]. In this study, we evaluated the reproductive biology and embryonic development of *Lysmata vittata* (Stimpson, 1860), an invader in the Atlantic Ocean.

*Lysmata vittata* is widely distributed in the Indian and Pacific oceans, where it occurs along the east coast of Africa, the coast of China, the Philippines, Japan, Indonesia and Australia [10–12]. This shrimp has also been reported to be an invader in New Zealand [13] and the coast of Brazil [14–16]. *Lysmata vittata* is commonly found at depths of 2 to 50 meters, living in large groups among rocks, algae, sponges, the octocoral *Carijoa riisei*, and other unidentified colonial cnidarians [10,11,15].

Various studies (e.g., [15,17–20] and references therein) suggest that all species in the genus *Lysmata* exhibit ‘protandric simultaneous hermaphroditism’ (PSH) (*sensu* [18]), an unusual sexual system in caridean shrimp [21]. In PSH, juveniles first mature as functional males (or MP—male-phase individuals), and later become functional simultaneous hermaphrodites (or FP—female-phase individuals) capable of reproducing as males or females [22]. Anatomical observations support that *L*. *vittata* is a protandric simultaneous hermaphrodite. The anatomical traits used to support such a suggestion are: 1) external sexual morphology; 2) internal anatomy (presence of ovotestes, i.e., paired gonads that show ovarian characteristics in the anterior part and testicular features in the posterior part, with two pairs of ducts corresponding to oviducts and vasa deferentia); and 3) a population size-frequency distribution of individuals in the size classes [15]. The same study emphasized the need for behavioral experiments in the laboratory in order to determine the functionality of *L*. *vittata* simultaneous hermaphrodites. Importantly, the sexual and mating system should be considered as another important species trait that may affect invasiveness [23,24]. However, the invading potential of a species depends not only its reproductive characteristics, but also on the morphological, life-history, and metabolic traits, which are related to the phenotypic plasticity of the species [25–27].

In this study, we were particularly interested in understanding the reproductive biology and embryonic development of *Lysmata vittata*. In this context, the objectives of this study were to evaluate: 1) the functionality of protandric simultaneous hermaphroditism (PSH); 2) the macro- and microscopic development of the ovarian portion of the ovotestes; 3) the reproductive cycle of laboratory-reared shrimp; and 4) embryonic development. In addition, we intended to provide important insights into the biology of invasion by comparing the reproductive traits of this shrimp with some of the predictions about aquatic invasive species (see above). To this end, with *L*. *vittata* being a successful invader, distributed widely, and in dense populations, within invaded areas ([13], present study), we should expect that this shrimp has 1) rapid development of the gonad, after it has been emptied, 2) short embryonic development, 3) short period between matings and, consequently, 4) a short reproductive cycle, with a high reproductive capacity.

## Material and methods

### Sampling of shrimp

**Fig 1:**
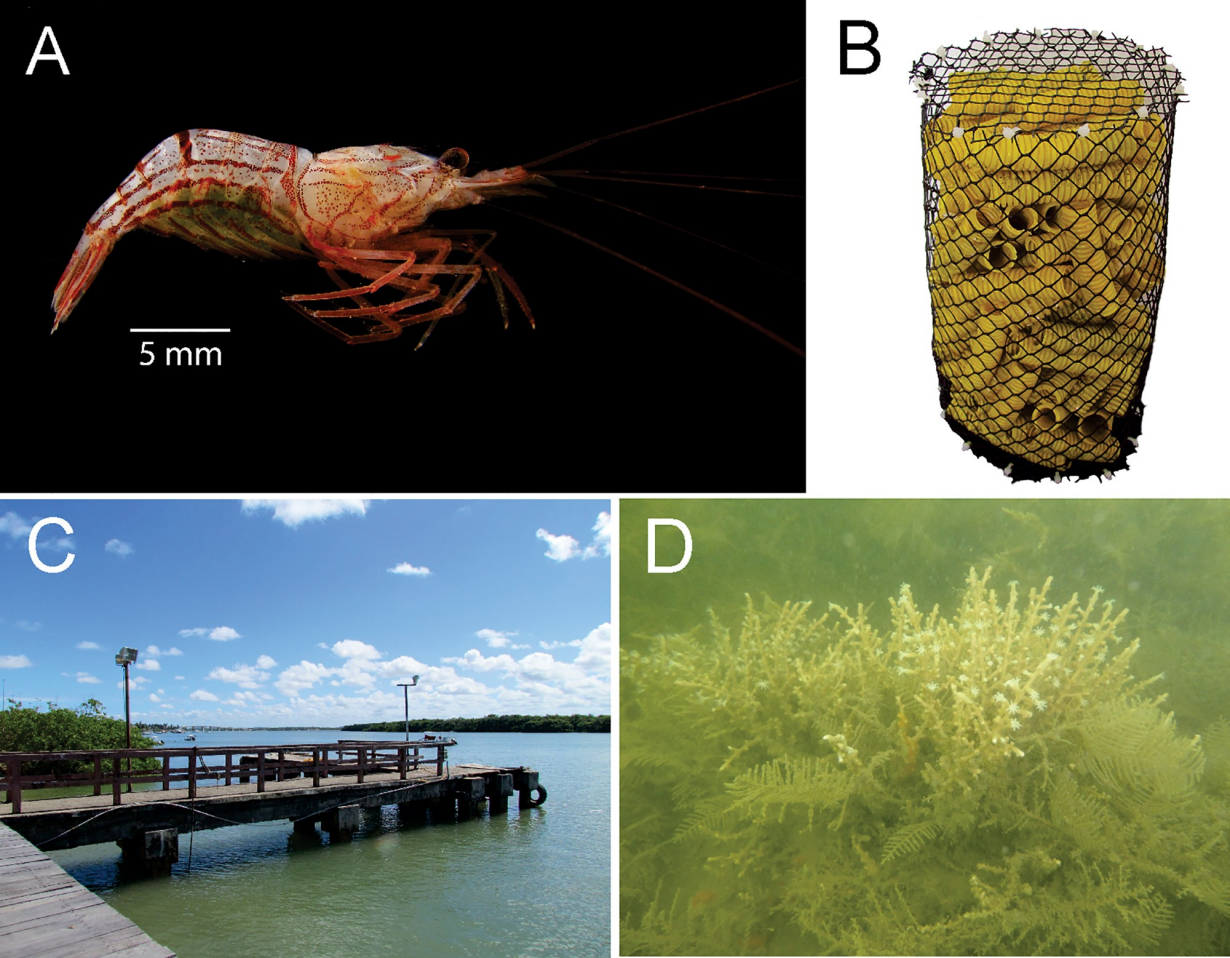
*Lysmata vittata* (Stimpson, 1860) and sampling sites. (A) Lateral view of ovigerous hermaphrodite. (B) Artificial Refuge Structures (ARS) used to obtain the shrimp. (C) Pier at Vaza-Barris estuary, Brazil. (D) Cnidarian polyps encrusting the pilasters. Photo credits: D.F.R. Alves.

Specimens of *L*. *vittata* (Fig 1A) were collected in the estuary region of the Vaza-Barris River, state of Sergipe, northeast of Brazil (11°05’59”S—37°08’59”W) from January 2015 to December 2016. Our study was permitted and supported by the Graduate Program in Ecology and Conservation of the University of Sergipe, Brazil (PNPD 20130610). This study did not involve endangered or protected species. For the collection of *L*. *vittata*, we built nine ‘Artificial Refuge Structures’ (ARS). Each of these ARS consisted of a plastic cylinder (15 cm diameter × 30 cm length) with 1 cm^2^ mesh, filled with flexible plastic tubes (1.8–2.4 cm diameter × 8 cm length) (Fig 1B). We installed the ARS on pilasters of a pier (Fig 1C), between 3 and 5 m deep, densely encrusted with *Thyroscyphus sp*. Allman, 1877, *Idiellana pristis* (Lamouroux, 1816) and *Carijoa riisei* (Duchassaing & Michelotti, 1860) (Fig 1D). After approximately 15 days, each ARS was recovered. Each ARS was placed into a net (500 μm mesh), and the structure was untied, and immediately taken to the pier, where the shrimp were manually removed from the ARS. The collected shrimp were individually placed into plastic bags with water from the collection site (200 ml). Shrimp were transported live in thermal boxes to the laboratory.

### Maintenance of shrimp in the laboratory

In the laboratory, shrimp were maintained in plastic tanks (120 × 70 × 120 mm), arranged in six aquaria (450 × 200 × 300 mm) connected to a water recirculation system [28]. The water recirculation system was equipped with a cooling device to ensure temperature control (accuracy of ± 0.5°C), a chemical and biological filter with a skimmer (Bubble Magus C7), an UV lamp (18 W), and activated carbon (500 g, replaced monthly).

All shrimp were maintained at a temperature of 26 ± 0.5°C, a salinity of 30 ± 1 PSU and a photoperiod of 12L:12D. The water used in the aquaria was prepared using tap water purified by a reverse osmosis/DI unit and synthetic sea salt suitable for a marine aquarium (Coral Pro Salt, Red Sea). Before experimental and laboratory observations, the carapace length (CL; distance from the post-orbital angle to the posterior margin of the carapace) of the shrimp was measured with a vernier caliper (accuracy 0.01 mm).

### Testing the functionality of protandric simultaneous hermaphroditism

In order to test the functionality of simultaneous hermaphroditism in *L*. *vittata*, each newly collected specimen was examined with the naked eye (if the ovarian portion of the gonad was visible), and the CL was measured. These observations were used to separate individuals in the male-phase (small body size, CL < 4.0 mm, and without a macroscopically visible ovarian portion of the gonad) from those in the female-phase (carrying embryos). During a previous study (see [14]) about 100 individual of *L*. *vittata* were collected in this same population and the smallest recorded hermaphrodite specimen measured 4.6 mm of carapace length. In the experiment, we used 12 shrimp in the male-phase and 18 shrimp in the female-phase. The shrimp were distributed to three treatments, with six replicates in each: Treatment 1, six pairs of shrimp carrying embryos (shrimp of larger sizes with mean ± SD = 4.73 ± 0.51 mm CL and with the ovarian portion of the ovoteste developed, each pair maintained in one rearing tank) to determine whether brooding hermaphroditic shrimp can simultaneously function as males; Treatment 2, six shrimp carrying embryos (with mean ± SD = 5.38 ± 0.71 mm CL, each shrimp maintained individually in one rearing tank) to verify if these shrimp produce a new brood without the presence of another shrimp, i.e., if the individual is capable of self-fertilization; and Treatment 3, six pairs of shrimp in the male-phase (with mean ± SD = 2.94 ± 0.37 mm CL, each pair maintained in one rearing tank), to verify if individuals who first matured as males later changed sex to become females. If, after successive molting, the shrimp in Treatment 3 grew, developed an ovarian portion of the ovotestes, and produced eggs, protandry would be confirmed. Otherwise, in Treatment 3, it was expected that the shrimp would remain as males, and, thus, individuals carrying eggs were not expected. Several laboratory conditions (e.g., nutritional value of the diet) may contribute to these shrimp remaining in the male-phase [29,30].

Twice a day, the shrimp were fed *ad libitum* with commercial food for ornamental fish (Thera—New Life Spectrum). In the morning, the shrimp were checked for the presence of eggs adhered to the pleopods and exuviae.

### Development of the ovarian portion of the ovotestes

After the sampling of brooding specimens of *L*. *vittata*, the size and coloration of the ovarian portion of the ovotestes were verified (by the naked eye) for the different stages of gonad development: developing ovary (DG), developed ovary (DE) and spent ovary (SP). We used a chromatic Pantone scale to characterize the gonad [31,32].

To validate the macroscopic examination of the development stage of the ovarian portion of the gonad, three individuals of each stage (DG, DE and SP) were analyzed microscopically. For the microscopic analysis, the shrimp were sacrificed after being cold-anesthetized at 5°C for 10 minutes, and their gonads were dissected under a stereomicroscope (Leica M205c), then fixed in Bouin’s solution for 8 hours at 25°C. The tissues were dehydrated and embedded in paraffin. Sections (5–7 μm thick) were stained with hematoxylin-eosin [33]. The sections were examined with a light microscope (Leica DM5500B) and photographs were taken with an imaging and measuring tool (Leica Application Suite–LAS, version 3.5.0). Subsequently, the largest diameter of 30 oogonia, primary oocytes and secondary oocytes were measured for each examined individual. The primary (or pre-vitellogenic) oocytes were identified by a homogeneous and acidophilic cytoplasm surrounded by round basophilic follicular cells. In contrast, secondary oocytes had an eosinophilic cytoplasm containing yolk droplets and globules, and were surrounded by flat and pyknotic follicular cells [34].

### Reproductive cycle of *Lysmata vittata*

Pairs of shrimp were observed for three consecutive reproductive cycles to estimate the mean duration (in days; mean ± SD) of three major events in the reproductive cycle: time to development of the ovarian portion of the gonad (TDO), recorded from the first day that the gonad was spent until the first day it could be seen as developed; time of embryonic development (TED), from the first day of new brood eggs to the day of hatched larvae; and time between broods (TBB), from the day of the hatched larvae until the day of the next brood of eggs. Twelve pairs of shrimp were used, each of which was composed of one male-phase individual (with mean ± SD = 3.49 ± 0.33 mm CL) and one female-phase individual (with mean ± SD = 5.23 ± 0.49 mm CL and carrying embryos). Each pair of shrimp was kept in plastic rearing tanks. Twice a day, the shrimp were fed *ad libitum* with commercial feed for ornamental fish (Thera—New Life Spectrum). The following measurements were recorded daily: 1) presence/absence of molt; 2) presence/absence of eggs adhered to the pleopods; and 3) stage of development of the ovarian portion of the gonad (DG, DE or SP), which was verified from macroscopic examinations of the gonad.

The model assumptions of homoscedasticity (Levene test) and normality (Shapiro-Wilks test) were initially tested [35]; when the results were unsatisfactory, we used nonparametric tests. To compare values obtained in each of the three reproductive cycles in relation to TDO, TED and TBB, a linear mixed effects (LME) analysis was used, due to the pseudo replication of plots in different reproductive cycles [36].

### Embryonic development of *Lysmata vittata*

Variation over the periods of embryonic development of the following characteristics were estimated: 1) egg volume; 2) percentage of the surface of the eggs completed by yolk; and 3) emergence of the main morphological structures of the embryos. Three pairs of shrimp were used, with each pair composed of one female-phase individual (mean ± SD = 5.58 ± 0.56 mm CL) and one individual in male-phase (mean ± SD = 3.43 ± 0.52 mm CL). The female-phase shrimp were collected in the wild with the ovarian portion of their gonads already developed. Each pair was maintained in a rearing tank and fed under the same conditions as previously described. Every morning, we recorded the presence/absence of eggs adhered to the pleopods.

From the day that a new brood was observed, embryo development was monitored every 24 hours. Five eggs from each shrimp were removed from the pleopods and immediately photographed under a stereomicroscope (Leica M205C) equipped with a multifocal imaging system. This procedure was repeated until the larvae release. From the images, the largest (L) and smallest (S) diameters of the eggs (elliptical shape) were measured. Egg volume (EV) was calculated using the formula EV = 1/6(LS^2^π) [37]. The total area of the egg and the area occupied by the yolk with embryos in the lateral position were measured using Leica Application Suite software. Finally, the day of emergence of the major morphological structures of the embryos was noted to describe the periods of embryonic development, according to standards used for other decapod crustaceans (e.g., [38,39]).

Only embryonic development that was completed in 8 days was described, since this time period was most frequently recorded during the reproductive cycle. To compare the volume of the eggs and the lateral surface area completed by the yolk in each periods of embryonic development, a linear mixed effects (LME) analysis was used, due to the pseudo replication of plots in different periods [36].

## Results

### Functionality of PSH

All specimens of *L*. *vittata* in Treatment 1 spawned and retained eggs that developed normally, which was recorded at least twice in less than 40 days. During the same period, none of the shrimp in Treatment 2 had a new brood. Furthermore, macroscopically, the development of the ovarian portion of the gonad was not verified in Treatment 3, and the brood was also not verified in this treatment.

### Development of the ovarian portion of the ovotestes

**Fig 2:**
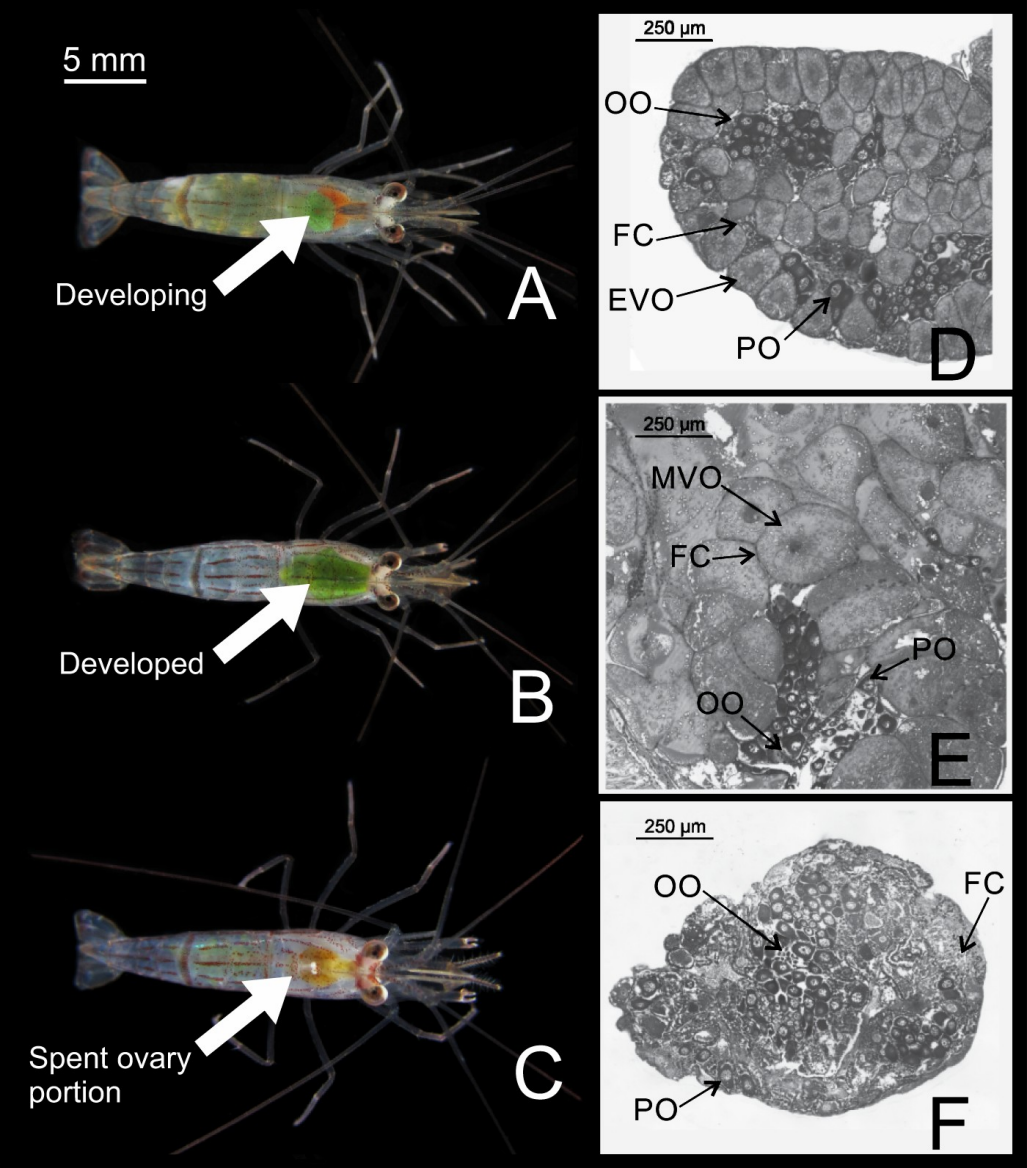
Development of the ovarian portion of the gonad in *Lysmata vittata* (Stimpson, 1860). (A–C) Stages identified macroscopically. (D) Developing ovary portion. (E) Developed ovary portion. (F) Spent ovary portion. EVO = early vitellogenesis oocyte (early secondary oocyte); FC = follicular cells; MVO = mature vitellogenesis oocyte (advanced secondary oocyte); OO = oogonia; PO = primary oocyte. Tissues were stained with hematoxylin-eosin.

Three stages of ovarian portion development (developing, developed and spent ovary) were distinguished using the naked eye (Fig 2A, 2B and 2C, S1 Table); this was confirmed by microscopic observations (Fig 2D, 2E and 2F). The three stages (macroscopic and microscopic) are described as follows:

#### Developing ovary

The ovarian portion of the gonad was macroscopically visible, occupying an area from the medial region of the carapace in dorsal view; light green coloration (Pantone 370M) (Fig 2A). Oogonia and primary oocytes (mean diameter ± SD = 47.7 ± 13.01 μm) were observed. Advanced oocytes in early vitellogenesis (mean diameter ± SD = 132.82 ± 31.51 μm) with yolk granules in the cytoplasm were also present (Fig 2D).

#### Developed ovary

The ovarian portion of the gonad was much larger than in previous stages, occupying most of the carapace in dorsal view; dark green coloration (Pantone 7496 M) (Fig 2B). Oogonia and primary oocytes (mean diameter ± SD = 41.21 ± 13.54 μm) were centrally observed. Many advanced oocytes (mean diameter ± SD = 232.24 ± 76.02 μm) with a large amount of yolk were also observed (Fig 2E).

#### Spent ovary

The gonad was difficult to recognize macroscopically; translucent (Fig 2C). Only oogonia, primary oocytes (mean diameter ± SD = 45.62 ± 12.07 μm) and many follicular cells were found in the central proliferation zone. The ovarian stroma appeared with some secondary oocytes undergoing resorption after laying (Fig 2F).

### Reproductive cycle of *Lysmata vittata*

Seventy days was sufficient time for all specimens (initially carrying embryos) to have three broods of eggs. Mating was always preceded by a molt event. The molt and mating occurred during the dark period of the photoperiod in all observations. In all observations, when the pre-copulatory exuvia was observed, the shrimp had eggs adhered to their pleopods, which developed normally into hatching larvae. In 16.7% (n = 6) of the observations, mating failed. In five of these observations, individual had unfertilized eggs (with different coloration), which were aborted and/or ingested by the parent. In one observation there was evidence of spawning, molting and spent ovary, but no eggs were found. In this case, eggs were aborted or ingested by the parent before the first observation.

**Fig 3:**
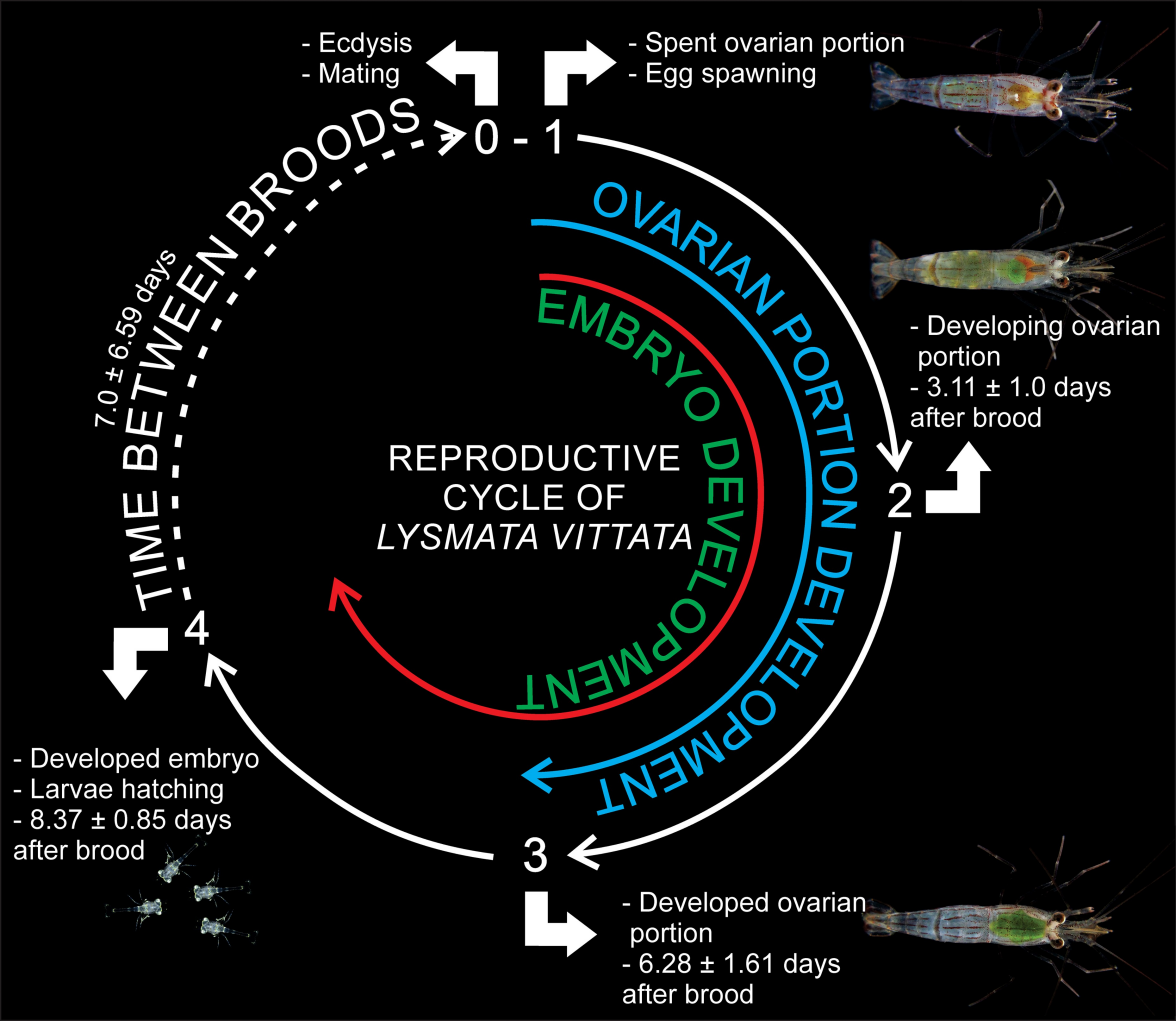
Reproductive cycle of *Lysmata vittata* (Stimpson, 1860) under laboratory conditions. The scheme shows the relationship among ecdysis, copulation, spawning and larvae hatching, based on the observation of 12 mating pairs for 70 days after three consecutive reproductive cycles.

Time for the development of the ovarian portion of the gonad (TDO) ranged from 4 to 11 days, with a mean (±SD) of 6.28 ± 1.61 days (Fig 3, S2 Table). The TDO did not differ significantly between the three reproductive cycles (Linear mixed effects model, F_1,23_ = 3.45, P = 0.08).

The time of embryonic development (TED) ranged from 7 to 11 days, with a mean (±SD) of 8.37 ± 0.85 days (Fig 3, S2 Table). The TED did not differ significantly between the three reproductive cycles (Linear mixed effects model, F_1,17_ = 1.94, P = 0.18). The brood of unfertilized eggs was observed on six occasions, which were aborted between 1 and 3 days after brooding.

The time between broods (TBB) ranged from 1 to 27 days, with a mean (±SD) of 7.00 ± 6.59 days (Fig 3, S3 Table). The mean time between broods (i.e., days between first and second reproductive cycle versus days between second and third reproductive cycle) did not differ significantly between the reproductive cycles (Linear mixed effects model, F_1,11_ = 0.01, P = 0.90).

### Embryonic development of *Lysmata vittata*

**Fig 4:**
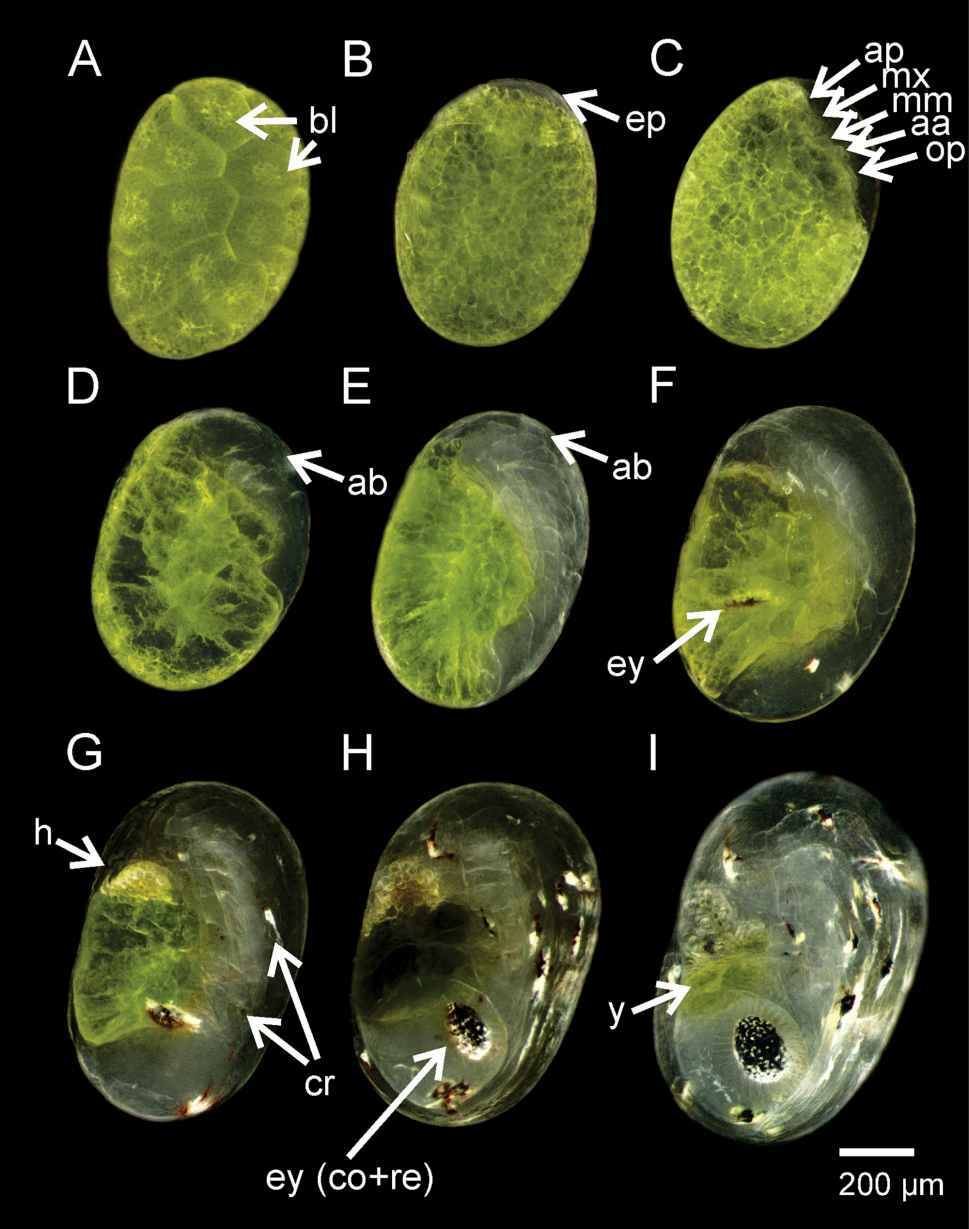
Embryonic development of *Lysmata vittata* (Stimpson, 1860). (A) Recently laid eggs. (B) Embryonic primordium. (C) Embryo with differentiated structures. (D) Abdomen and cephalothorax are differentiated. (E) Abdomen is enlarged and its segmentation is almost complete. (F) Eyes are developing. (G) Appearance of chromatophores. (H) Eyes are differentiated into cornea and retina. (I) Embryo is close to hatching. aa = antennule-antenna lobe; ab = abdomen; ap = abdomen primordium; bl = blastomere; cr = chromatophores; ep = embryonic primordium; ey = eye; ey (cornea and retina) = eye is differentiated into cornea and retina; h = heart; mm = maxillule-maxilla lobe; mx = maxilliped lobe; op = optic lobe; y = yolk.

The embryonic development of *L*. *vittata* can be divided into nine periods (P) (Fig 4, S4 Table) based on the differentiation of segmentation (P1, Fig 4A), the appearance of embryonic primordium (P2, Fig 4B), the appearance of lobes of the major embryo structures (P3, Fig 4C), the development of the abdomen (P4, Fig 4D), the abdomen being enlarged and having almost complete segmentation (P5, Fig 4E), the appearance of pigmentation in the optic lobes (P6, Fig 4F), differentiation of chromatophores in the mouth and abdominal region (P7, Fig 4G), differentiation of the cornea and retina in the eyes (P8, Fig 4H) and the embryo being close to hatching, with a small amount of yolk droplets in the dorsal region of the cephalothorax (P9, Fig 4I). The embryonic developmental periods of *L*. *vittata* are described as follows:

#### Period 1

Recently laid eggs. The eggs are macrolecithal-centrolecithal and spherical, with a mean volume (± SD) of 0.109 ± 0.01 mm^3^ and a green yolk mass occupying 100% of the egg volume. No evidence of an embryo (Fig 4A).

#### Period 2

One day after laying eggs. The eggs have a mean volume (± SD) of 0.103 ± 0.01 mm^3^. The yolk is fragmented into small oily droplets. Yolk mass is green in color, occupying 94 ± 3% of the egg volume. Cells are organized in one of the poles of the egg, initiating embryonic differentiation (Fig 4B).

#### Period 3

Two days after laying eggs. The eggs have a mean volume (± SD) of 0.112 ± 0.01 mm^3^. The yolk mass occupies 84 ± 6% of the egg volume. The cluster of primordial cells has differentiated into major embryo structures: ocular, antennule-antenna, maxillule-maxilla, maxilliped and thoracic-abdominal (Fig 4C).

#### Period 4

Three days after laying eggs. The eggs have a mean volume (± SD) of 0.121 ± 0.01 mm^3^. The yolk mass occupies 82 ± 6% of the egg volume. Abdominal and cephalothoracic primordia have increased in size and are now differentiated. Antennule-antenna, maxillule-maxilla and maxillipeds are seen as tiny buds rising below and behind the optical primordial structures (Fig 4D).

#### Period 5

Four days after laying eggs. The eggs have a mean volume (± SD) of 0.139 ± 0.01 mm^3^. The yolk mass occupies 71 ± 8% of the egg volume. Antennule-antenna, maxillule-maxilla and maxilliped primordia have grown. The abdomen is enlarged and its segmentation is almost complete (metameres) (Fig 4E).

#### Period 6

Five days after laying eggs. The eggs have a mean volume (± SD) of 0.155 ± 0.01 mm^3^ and the yolk mass occupies 51 ± 6% of the egg volume. The cephalothorax is formed. Eyes are developing into a slightly darker oval shaped core (retina) in the anterior portion of the embryo. All appendages are segmented and enlarged (Fig 4F).

#### Period 7

Six days after laying eggs. The eggs have a mean volume (± SD) of 0.171 ± 0.01 mm^3^. The yolk mass occupies 37 ± 7% of the egg volume. Yolk droplets are still stored in the dorsal portion of the cephalothorax. Chromatophores are visible in the appendices and abdomen. The heart has grown and is beating. Eyes are enlarged, rounded and densely pigmented (Fig 4G).

#### Period 8

Seven days after laying eggs. The eggs have a mean volume of 0.192 ± 0.01 mm^3^. The yolk mass occupies 23 ± 4% of the egg volume. The abdomen shows a pair of chromatophores on each segment. The eyes are differentiated in the cornea and retina (Fig 4H).

#### Period 9

**Fig 5:**
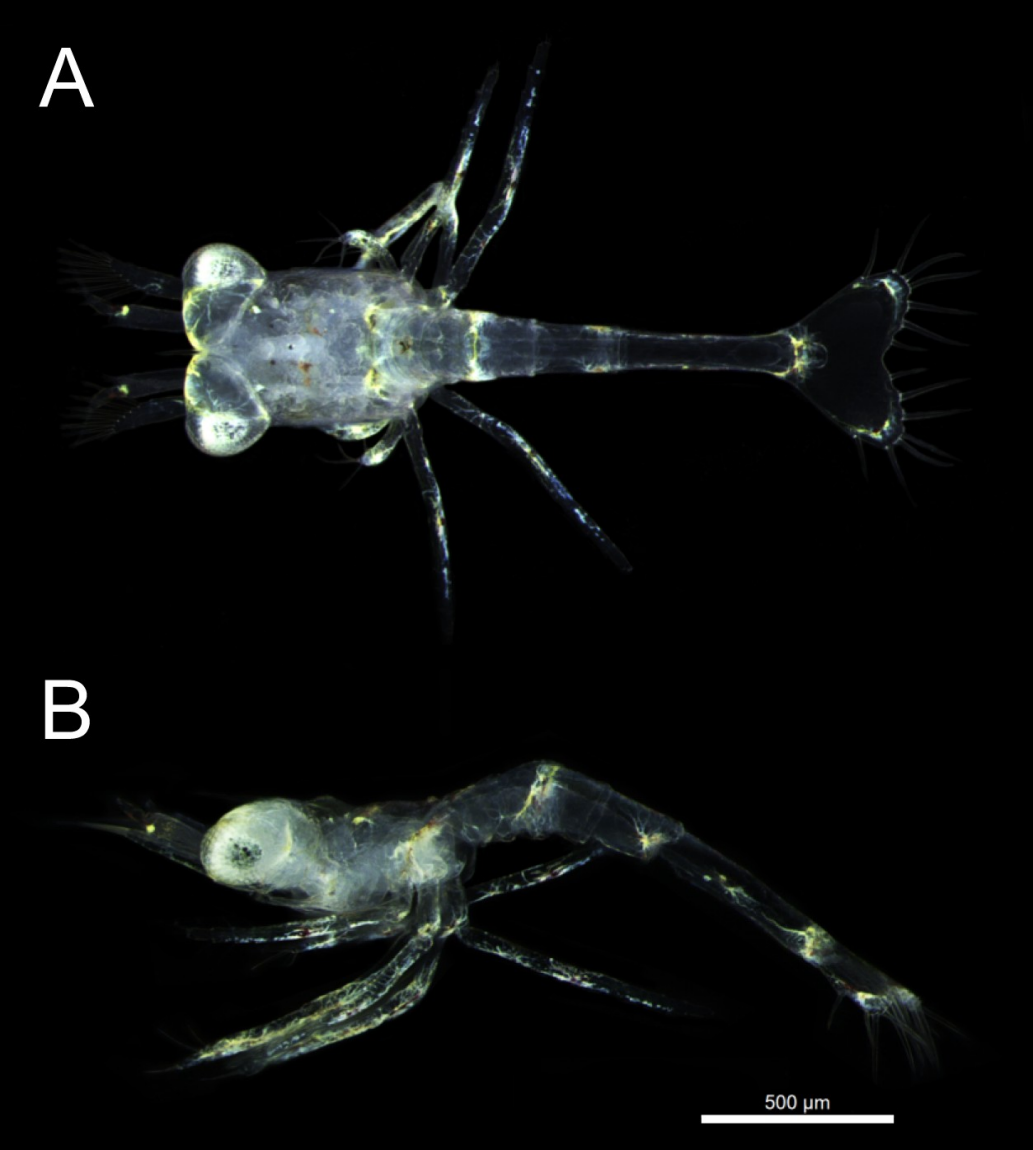
Newly hatched *Lysmata vittata* (Stimpson, 1860) zoea I. (A) Dorsal view. (B) Lateral view.

Eight days after laying eggs. The eggs have a mean volume (± SD) of 0.229 ± 0.01 mm^3^. A few small yolk droplets remain dorsally in the cephalotorax. The yolk mass occupies 7 ± 2% of the egg volume. The embryo is close to hatching (Fig 4I). Larvae (zoea I) commonly hatch (Fig 5) in the late afternoon or early evening, without a remnant of the yolk reserve.

**Fig 6:**
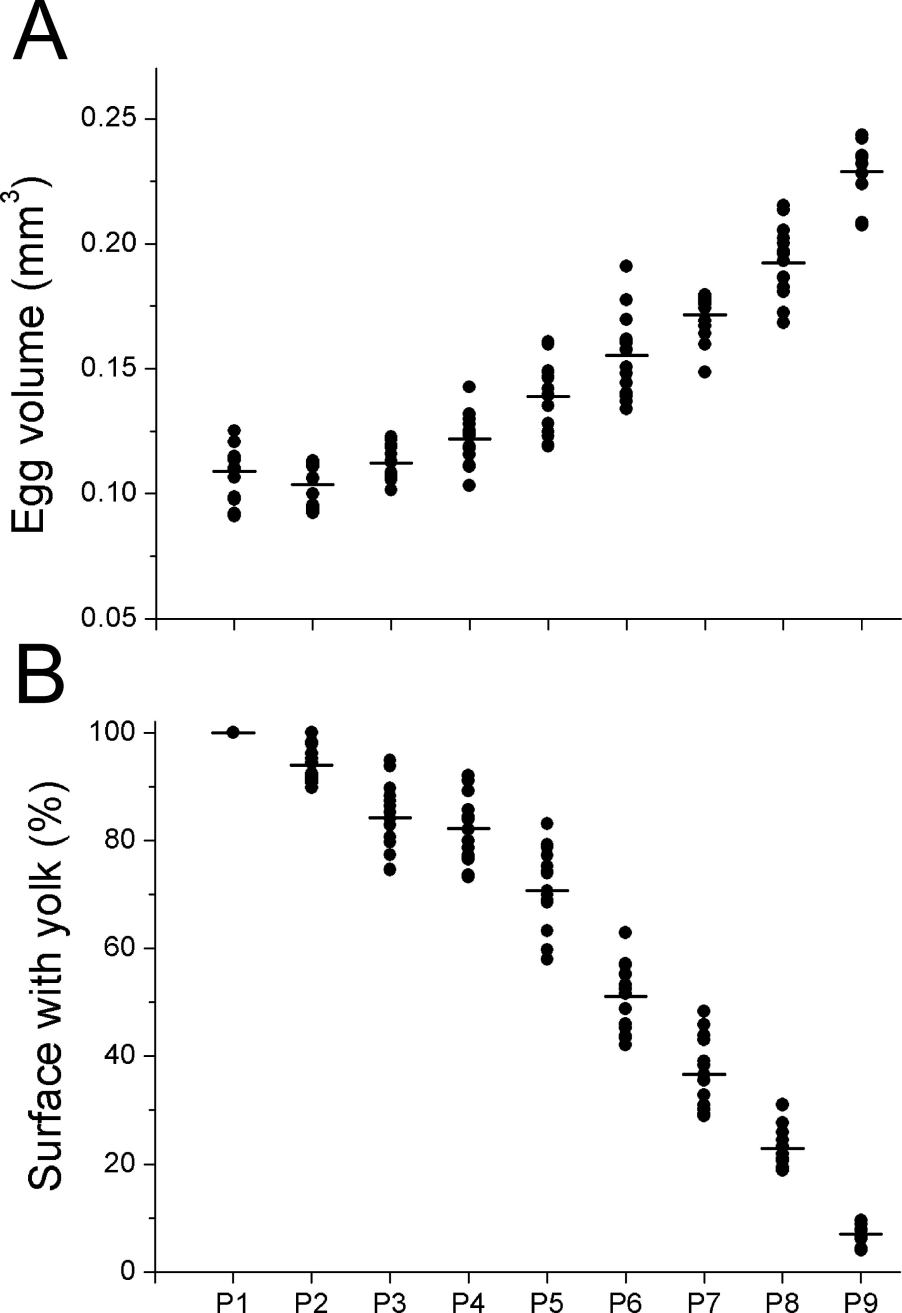
*Lysmata vittata* (Stimpson, 1860) eggs in different periods (P) of embryonic development. (A) Egg volume (mm^3^). (B) Surface with yolk (%). Bars (―) indicate mean values.

Mean egg volume increased throughout embryonic development and ranged significantly between periods (Linear mixed effects model, F_1,123_ = 1588.11, P < 0.001, Fig 6A). Moreover, as the embryo developed, the lateral surface area of the egg occupied by the yolk decreased rapidly, with significant differences between periods (Linear mixed effects model, F_1,123_ = 168.90, P < 0.001, Fig 6B).

## Discussion

### Reproductive traits and embryonic development of *L*. *vittata*

The experiment on the functionality of PSH demonstrated that brooding shrimp are able to fertilize each other and do not self-fertilize. First, brooding individuals maintained in isolation retained a developed ovarian portion of the gonad until the end of the experiment, thus, these shrimp did not produce eggs. In two out of six shrimp, the gonad returned to the spent condition, but we did not observe the adherence of any eggs in the shrimp pleopods. Unlike the results recorded here for *L*. *vittata*, in a similar experiment, all *L*. *nayaritensis* shrimp produced eggs, but none had developing embryos (see [40]). Second, individuals of *L*. *vittata* can act as males even when the shrimp have a visible ovarian portion of the ovotestes and/or carry embryos in development. This demonstrates that the shrimp can act simultaneously as males and females. Lastly, the smallest specimens of *L*. *vittata* only act as males and, during the experimental period, these small male specimens did not develop the ovarian portion of the gonad and did not produce eggs. Therefore, observations from this experiment demonstrate the functionality of simultaneous hermaphroditism in *L*. *vittata*, as reported for other studied species of the genus [17,18,22,41,42]. This observation supports the hypothesis that protandric simultaneous hermaphroditism is a conserved trait within the *Lysmata* genus [19]. However, further laboratory experimentation is necessary to test the functionality of this sexual system in other species of the *Lysmata* genus; such information can be used to generate better comparative analyses of the evolutionary origins and adaptive value of this unusual sexual system.

In addition, studying the reproductive cycle of *L*. *vittata*, we also verified that larvae hatch and shrimp present the ovarian portion of the developed ovotestes an average of 8 days after copula. Mating is always preceded by molting, which is common in caridean and stenopodidean shrimp [43–45]. We also verified that the period between successive broods is short (1 or 2 days in most observations). However, in some cases the copula failed, i.e., an exuvia was verified and the ovarian portion returned to the spent condition, but we did not observe eggs adhering in the shrimp pleopods. In these cases, the period between successive broods was longer (up to 27 days). The factors that may negatively affect the copula for *Lysmata* shrimp in culture systems are scarcely known. Thus, specific studies of shrimp copula in culture systems are necessary.

The reproductive cycle of *L*. *vittata* occurred over a short period, compared to previous studies with other congeners (*Lysmata amboinensis* > 12 days [17,46]; *L*. *wurdemanni* > 11 days [47]). This result suggests that *L*. *vittata* has a high reproductive capacity in a short period of time. In this study, we controlled the temperature at which the shrimp were maintained in the laboratory, based on the average recorded temperature in the wild, since temperature is commonly related to time of embryonic development in decapod crustaceans [48,49].

The analysis of the reproductive cycle was made possible by the use of histological examinations, which confirmed macroscopic observation of three stages of development in the ovarian portion of the ovotestes. This macroscopic identification was possible since the ovarian portion of the *L*. *vittata* gonad varies as it develops, in terms of the area that it occupies in the cephalothorax in dorsal view. Moreover, the color of this gonad is distinct in the three stages of development (light green, dark green and translucent). Macroscopic differences are due to an increase in the number of primary oocytes and in the size of these cells throughout ovarian development, which has been attributed to the deposit of lipids during vitellogenesis. This pattern of ovarian development has previously been described for other shrimp species (*Macrobrachium rosenbergii* [50]; *Macrobrachium amazonicum* [51]; *Stenopus hispidus* [32]; *Plesionika edwardsii* [52]). On the other hand, for other shrimp species, the carapace is slightly translucent and macroscopic examination is therefore difficult and/or impossible without sacrificing the specimen.

The embryonic development of *L*. *vittata* was unknown until the present study. In the genus *Lysmata*, only the embryonic development of *Lysmata boggessi* was previously described [53]. Thus, the embryonic development of most species of *Lysmata* has not been examined. These studies are needed, given that these shrimp exhibit a wide variety of lifestyles, mating systems, social behaviors and symbiotic partnerships [20]. According to the results of this study, *L*. *vittata* follow the standard caridean pattern of early development, with early holoblastic cleavage [53–55]. The time required for complete embryonic development up to larvae hatching in *L*. *vittata* seems to be the lowest (~ 8 days) compared with other previous studied species of the *Lysmata* genus (e.g., 10 to 12 days for *L*. *amboinensis*, *L*. *boggessi* and *L*. *debelius* [17,53,56]). Moreover, rapid embryonic development favors a fast reproductive cycle for this shrimp.

We also found that *L*. *vittata* larvae hatch without macroscopically visible yolk reserves. This result suggests that *L*. *vittata* larvae have a high dependence on exogenous food after hatching. However, unpublished experimental observations indicate that *L*. *vittata* larvae have a high resistance to starvation in the first days after hatching (DF, personal observation). Similarly, the absence of a dependence on exogenous food for early *L*. *boggessi* larvae was verified experimentally [57]. In this species, the yolk was distributed in about 10–20% of the cephalothorax in newly hatched larvae [53].

### Do the reproductive characteristics and the development of *L*. *vittata* favor the invasion of new territories?

In this study, we used some aspects of *L*. *vittata* reproductive biology and development of in order to test some predictions about aquatic invasive species, i.e., that this species has a short generation time and a high reproductive capacity [9]. In general, our observations and laboratory data about *L*. *vittata* reproductive and developmental characteristics are in favor of the predictions mentioned above. First, *L*. *vittata* has a rapid embryonic development and rapid development of the ovarian portion of the ovotestes, and, consequently, a short reproductive cycle.

Second, the confirmation that *L*. *vittata* adopts protandric simultaneous hermaphroditism as a sexual system is also relevant in the context of these shrimp invading new territories. In this case, the self-fertilization would favor initial invasion [24], but our results indicate that *L*. *vittata* is not able to self-fertilize. On the other hand, studies have suggested that outcrossing reproduction favors genetic variability [58], being this a common attribute of successful invasive species [9,24]. Thus, as *L*. *vittata* is a protandric simultaneous hermaphrodite, any two individuals (or small groups) introduced to a new region could potentially establish a new population.

Lastly, in addition to environmental factors that may affect larval survival (e.g., salinity, temperature, food availability), the time required for the development of planktonic decapods is another relevant factor to the invasion of new territories. The dissemination patterns of populations of sessile or sedentary marine organisms are directly related to their dispersal ability [59]. Moreover, decapod crustaceans disperse more commonly during the planktonic larval stages, naturally as well as during invasions. In this context, the development of the nine larval stages of *L*. *vittata* has a duration ranging from 27 to 45 days [60]. Therefore, *L*. *vittata* continues to develop as plankton for a long time, compared to other invasive decapods, such as *Petrolisthes armatus* (Gibbes, 1850) (12 to 19 days as planktonic larvae) [61]. Thus, time in the plankton stage is another factor that must be considered with respect to the invasion success of *L*. *vittata*.

Our results provide indications that the invasive shrimp *L*. *vittata* have reproductive and embryonic development characteristics that may be favorable to the establishment of populations during invasive processes. Thus, the results of the present study are in accordance with the predictions regarding the characteristics of invasive aquatic species.

### Outlook

Studies that evaluate potentially favorable characteristics of species for success during invasions are necessary. For *Lysmata vittata* shrimp, studies are still needed to evaluate: 1) the average time for individuals to become sexually mature, first as males and later as simultaneous hermaphrodites; 2) the tolerance to variation in environmental factors (e.g., salinity, temperature, food availability) for larvae and post-larval stages; 3) the somatic growth rate. Besides *L*. *vittata*, several species of decapod crustaceans are considered invasive in the western Atlantic Ocean, e.g., *Bellia picta* H. Milne Edwards, 1848; *Cancer pagurus* Linnaeus, 1758; *Halicarcinus planatus* (Fabricius, 1775); *Liocarcinus navigator* (Herbst, 1794); *Metapenaeus monoceros* (Fabricius, 1798); *Pilumnoides perlatus* (Poeppig, 1836); *Scylla serrata* (Forskål, 1775); and *Taliepus dentatus* (H. Milne Edwards, 1834) [62]. In this context, the following question remains: what are the characteristics that favored the invasion of each of these species in the western Atlantic Ocean? Finally, with the knowledge of such biological characteristics, it is possible to create and/or improve the predictive models of areas susceptible to new invasions by each species. Thus, information on the characteristics of invasive species can be used to formulate important tools for the conservation of biological diversity.

## Supporting information

S1 Table Development of the ovarian portion of the gonad in *Lysmata vittata* (Stimpson, 1860). Legend: Minimum (Min), maximum (Max) and mean ± standard deviation (X ± DP) diameter size (μm) of primary and advanced oocytes in different stages of development of the gonad (developing, developed and spent).
(PDF)

S2 Table Reproductive cycle of *Lysmata vittata* (Stimpson, 1860) under laboratory conditions. Time (in days) for the development of the ovarian portion of the gonad (TDO) and time (in days) of embryonic development (TED) for each replicate in different reproductive cycle (1, 2, and 3).
(PDF)

S3 Table Reproductive cycle of *Lysmata vittata* (Stimpson, 1860) under laboratory conditions. Time (in days) between broods (TBB) for each replicate in different reproductive cycle (1–2, and 2–3).
(PDF)

S4 Table Embryonic development of *Lysmata vittata* (Stimpson, 1860). Volume and area of the egg, yolk area of the egg and percentage (%) of the area of the egg occupied of yolk per replicate in each period of the embryonic development.
(PDF)
