# Supplementary material for: Sexual system, reproductive cycle and embryonic development of the red-striped shrimp Lysmata vittata, an invader in the western Atlantic Ocean
Source: PLoS One. 2019 Jan 15;14(1):e0210723. doi: 10.1371/journal.pone.0210723 (PMC6333369; doi:10.1371/journal.pone.0210723)
Supplement: S1 Table — Legend: Minimum (Min), maximum (Max) and mean ± standard deviation (X ± DP) diameter size (μm) of primary and advanced oocytes in different stages of development of the gonad (developing, developed and spent). (PDF) [file pone.0210723.s001.pdf]

S1 Table

	Primary oocytes			Advanced oocytes		
	**Min**	**Max**	**X ± DP**	**Min**	**Max**	**X ± DP**
Developing	20.76	84.18	47.7 ± 13.01	54.50	190.32	132.82 ± 31.51
Developed	15.05	73.83	41.21 ± 13.54	110.75	394.92	232.24 ± 76.02
Spent	24.18	88.17	45.62 ± 12.07	-	-	-
