# Supplementary material for: Sexual system, reproductive cycle and embryonic development of the red-striped shrimp Lysmata vittata, an invader in the western Atlantic Ocean
Source: PLoS One. 2019 Jan 15;14(1):e0210723. doi: 10.1371/journal.pone.0210723 (PMC6333369; doi:10.1371/journal.pone.0210723)
Supplement: S2 Table — Time (in days) for the development of the ovarian portion of the gonad (TDO) and time (in days) of embryonic development (TED) for each replicate in different reproductive cycle (1, 2, and 3). (PDF) [file pone.0210723.s002.pdf]

S2 Table

Replicate	Cycle reproductive	TDO (in days)	TED (in days)
**1**	1	6	9
**2**	1	8	9
**3**	1	6	10
**4**	1	6	9
**5**	1	9	8
**6**	1	5	9
**7**	1	8	7
**8**	1	6	7
**9**	1	9	8
**10**	1	6	9
**11**	1	6	9
**12**	1	6	11
**1**	2	5	8
**2**	2	9	na
**3**	2	5	8
**4**	2	5	8
**5**	2	7	8
**6**	2	5	8
**7**	2	11	7
**8**	2	6	na
**9**	2	6	8
**10**	2	4	na
**11**	2	5	8
**12**	2	8	na
**1**	3	6	8
**2**	3	8	8
**3**	3	5	9
**4**	3	4	9
**5**	3	5	8
**6**	3	5	na
**7**	3	5	8
**8**	3	8	9
**9**	3	5	8
**10**	3	5	8
**11**	3	6	na
**12**	3	7	8
