# Supplementary material for: Sexual system, reproductive cycle and embryonic development of the red-striped shrimp Lysmata vittata, an invader in the western Atlantic Ocean
Source: PLoS One. 2019 Jan 15;14(1):e0210723. doi: 10.1371/journal.pone.0210723 (PMC6333369; doi:10.1371/journal.pone.0210723)
Supplement: S3 Table — Time (in days) between broods (TBB) for each replicate in different reproductive cycle (1–2, and 2–3). (PDF) [file pone.0210723.s003.pdf]

S3 Table

**Replicate**	**Cycle reproductive**	**TBB (in days)**
**1**	1-2	1
**2**	1-2	2
**3**	1-2	10
**4**	1-2	9
**5**	1-2	3
**6**	1-2	3
**7**	1-2	1
**8**	1-2	10
**9**	1-2	27
**10**	1-2	7
**11**	1-2	2
**12**	1-2	11
**1**	2-3	2
**2**	2-3	12
**3**	2-3	1
**4**	2-3	14
**5**	2-3	2
**6**	2-3	4
**7**	2-3	10
**8**	2-3	20
**9**	2-3	2
**10**	2-3	7
**11**	2-3	1
**12**	2-3	7
