# Supplementary material for: Sexual system, reproductive cycle and embryonic development of the red-striped shrimp Lysmata vittata, an invader in the western Atlantic Ocean
Source: PLoS One. 2019 Jan 15;14(1):e0210723. doi: 10.1371/journal.pone.0210723 (PMC6333369; doi:10.1371/journal.pone.0210723)
Supplement: S4 Table — Volume and area of the egg, yolk area of the egg and percentage (%) of the area of the egg occupied of yolk per replicate in each period of the embryonic development. (PDF) [file pone.0210723.s004.pdf]

S4 Table

Replicate	Period	Volume (mm^3)	Area (mm^3)	Yolk area (mm^3)	Yolk (%)
**1**	P1	0.092	0.277	0.277	100.00
**1**	P1	0.099	0.294	0.294	100.00
**1**	P1	0.091	0.278	0.278	100.00
**1**	P1	0.107	0.314	0.314	100.00
**1**	P1	0.098	0.292	0.292	100.00
**1**	P2	0.100	0.305	0.281	92.13
**1**	P2	0.096	0.306	0.289	94.44
**1**	P2	0.092	0.303	0.272	89.77
**1**	P2	0.106	0.286	0.263	91.96
**1**	P2	0.093	0.278	0.257	92.45
**1**	P3	0.106	0.300	0.269	89.67
**1**	P3	0.106	0.316	0.262	82.91
**1**	P3	0.102	0.301	0.260	86.38
**1**	P3	0.107	0.304	0.259	85.20
**1**	P3	0.108	0.318	0.267	83.96
**1**	P4	0.111	0.340	0.303	89.12
**1**	P4	0.103	0.302	0.278	92.05
**1**	P4	0.125	0.336	0.288	85.71
**1**	P4	0.119	0.312	0.284	91.03
**1**	P4	0.111	0.325	0.290	89.23
**1**	P5	0.119	0.344	0.254	73.84
**1**	P5	0.119	0.358	0.282	78.77
**1**	P5	0.128	0.347	0.268	77.23
**1**	P5	0.125	0.359	0.270	75.21
**1**	P5	0.123	0.355	0.212	59.72
**1**	P6	0.140	0.376	0.197	52.39
**1**	P6	0.134	0.366	0.154	42.08
**1**	P6	0.139	0.376	0.194	51.60
**1**	P6	0.137	0.386	0.203	52.59
**1**	P6	0.144	0.375	0.164	43.73
**1**	P7	0.149	0.425	0.151	35.53
**1**	P7	0.167	0.452	0.198	43.81
**1**	P7	0.178	0.467	0.141	30.19
**1**	P7	0.176	0.445	0.174	39.10
**1**	P8	0.181	0.482	0.133	27.59
**1**	P8	0.186	0.494	0.108	21.86
**1**	P8	0.193	0.490	0.095	19.39
**1**	P8	0.197	0.497	0.116	23.34
**1**	P8	0.205	0.516	0.097	18.80
**1**	P9	0.234	0.569	0.025	4.39
**1**	P9	0.209	0.533	0.022	4.13
**1**	P9	0.207	0.509	0.031	6.09
**1**	P9	0.224	0.528	0.034	6.44
**1**	P9	0.232	0.553	0.022	3.98
**2**	P1	0.115	0.325	0.325	100.00
**2**	P1	0.114	0.322	0.322	100.00

**2**	P1	0.114	0.324	0.324	100.00
**2**	P1	0.113	0.335	0.335	100.00
**2**	P1	0.109	0.314	0.314	100.00
**2**	P2	0.113	0.335	0.316	94.33
**2**	P2	0.106	0.314	0.286	91.08
**2**	P2	0.111	0.317	0.290	91.48
**2**	P2	0.106	0.313	0.284	90.73
**2**	P2	0.113	0.326	0.301	92.33
**2**	P3	0.116	0.327	0.244	74.62
**2**	P3	0.120	0.318	0.237	74.53
**2**	P3	0.108	0.320	0.255	79.69
**2**	P3	0.113	0.310	0.250	80.65
**2**	P3	0.109	0.313	0.242	77.32
**2**	P4	0.123	0.348	0.292	83.91
**2**	P4	0.143	0.370	0.283	76.49
**2**	P4	0.118	0.329	0.270	82.07
**2**	P4	0.116	0.320	0.270	84.38
**2**	P4	0.119	0.329	0.263	79.94
**2**	P5	0.147	0.375	0.297	79.20
**2**	P5	0.140	0.373	0.277	74.26
**2**	P5	0.142	0.378	0.314	83.07
**2**	P5	0.139	0.367	0.232	63.22
**2**	P5	0.135	0.366	0.251	68.58
**2**	P6	0.162	0.408	0.199	48.77
**2**	P6	0.151	0.406	0.231	56.90
**2**	P6	0.148	0.387	0.221	57.11
**2**	P6	0.158	0.409	0.257	62.84
**2**	P6	0.158	0.404	0.183	45.30
**2**	P7	0.164	0.447	0.205	45.86
**2**	P7	0.178	0.460	0.168	36.52
**2**	P7	0.169	0.416	0.179	43.03
**2**	P7	0.176	0.458	0.221	48.25
**2**	P7	0.178	0.449	0.172	38.31
**2**	P8	0.172	0.476	0.123	25.84
**2**	P8	0.200	0.502	0.103	20.52
**2**	P8	0.202	0.509	0.097	19.06
**2**	P8	0.183	0.498	0.104	20.88
**2**	P8	0.187	0.499	0.122	24.45
**3**	P1	0.114	0.320	0.320	100.00
**3**	P1	0.114	0.319	0.319	100.00
**3**	P1	0.125	0.342	0.342	100.00
**3**	P1	0.121	0.337	0.337	100.00
**3**	P1	0.110	0.316	0.316	100.00
**3**	P2	0.112	0.329	0.329	100.00
**3**	P2	0.111	0.322	0.315	97.83
**3**	P2	0.094	0.292	0.278	95.21
**3**	P2	0.106	0.312	0.300	96.15
**3**	P2	0.094	0.284	0.279	98.24
**3**	P3	0.123	0.326	0.309	94.79
**3**	P3	0.119	0.353	0.331	93.77

**3**	P3	0.113	0.316	0.279	88.29
**3**	P3	0.122	0.339	0.296	87.32
**3**	P4	0.124	0.346	0.272	78.61
**3**	P4	0.128	0.352	0.259	73.58
**3**	P4	0.130	0.362	0.265	73.20
**3**	P4	0.124	0.343	0.263	76.68
**3**	P4	0.132	0.352	0.272	77.27
**3**	P5	0.146	0.419	0.293	69.93
**3**	P5	0.149	0.397	0.274	69.02
**3**	P5	0.161	0.419	0.243	58.00
**3**	P5	0.160	0.414	0.247	59.66
**3**	P5	0.149	0.395	0.279	70.63
**3**	P6	0.177	0.459	0.211	45.97
**3**	P6	0.170	0.426	0.235	55.16
**3**	P6	0.161	0.429	0.237	55.24
**3**	P6	0.160	0.434	0.231	53.23
**3**	P6	0.191	0.466	0.202	43.35
**3**	P7	0.160	0.402	0.121	30.10
**3**	P7	0.177	0.448	0.147	32.81
**3**	P7	0.177	0.451	0.132	29.27
**3**	P7	0.174	0.452	0.131	28.98
**3**	P7	0.180	0.443	0.137	30.93
**3**	P8	0.196	0.496	0.096	19.35
**3**	P8	0.215	0.506	0.098	19.37
**3**	P8	0.213	0.508	0.107	21.06
**3**	P8	0.168	0.436	0.135	30.96
**3**	P8	0.186	0.486	0.151	31.07
**3**	P9	0.244	0.585	0.046	7.86
**3**	P9	0.242	0.576	0.055	9.55
**3**	P9	0.228	0.553	0.042	7.59
**3**	P9	0.235	0.567	0.050	8.82
**3**	P9	0.233	0.575	0.064	11.13
